# Atypical Giant Suprasellar Prolactinoma Presenting With Visual Field Changes in the Absence of Symptoms of Hyperprolactinemia

**DOI:** 10.7759/cureus.19632

**Published:** 2021-11-16

**Authors:** Scott Sun, Jun Q Mo, Michael L Levy, John Crawford

**Affiliations:** 1 Medicine, University of California San Diego, San Diego, USA; 2 Pathology, University of California San Diego, San Diego, USA; 3 Neurosurgery, University of California San Diego, San Diego, USA; 4 Neurosciences and Pediatrics, University of California San Diego, San Diego, USA

**Keywords:** prolactinoma, pituitary adenoma, suprasellar tumor, visual field, atypical neuroimaging

## Abstract

Prolactinomas are benign tumors that make up the majority of all pituitary adenoma cases and present most commonly in women. Prolactinomas presenting in adolescents and children, however, are extremely rare. We report a case of a 17-year-old male who presented with a six-month history of headaches and a previously unrecognized visual field deficit on examination. Neuroimaging revealed a large suprasellar tumor with imaging, more characteristic of a craniopharyngioma or suprasellar low-grade glioma impinging, on the left intracranial optic nerve causing right-sided hemianopsia. Due to the extensive mass effect and bitemporal hemianopsia on examination, the decision to proceed with initial surgical debulking was made following informed consent. A subtotal resection was performed where the pathology was consistent with a prolactinoma that correlated with markedly elevated prolactin (PRL) levels obtained pre and post-operatively that have not resulted until five days post procedure.

The patient was subsequently treated with dopamine agonist (DA) cabergoline therapy and is now five-years disease-free with normal neurological examination and no residual tumor on neuroimaging. DA therapy has shown high clinical efficacy and should be considered prior to any surgical intervention; however, extensive mass effect may appropriate surgical debulking to increase therapy efficacy. Our case highlights an atypical appearance of a giant prolactinoma that may mimic other more common suprasellar tumors, a presentation associated with unrecognized visual field deficits, and the importance of rapid turnaround testing for serum PRL that may aid in the upfront diagnosis and management of prolactinomas.

## Introduction

The pituitary gland plays a critical role in the maintenance of endocrinological balance with specific regard to its development, reproduction, and growth. Adenomas impinging on the pituitary can lead to significant pituitarism, such as hyperprolactinemia caused by prolactinomas [1]. Despite being the most common type of pituitary tumor, prolactinomas are exceedingly rare in the children and adolescent demographic group, presenting in less than 2% of all intracranial tumor cases [2]. The potential role of surgery in the upfront management of prolactinomas is not entirely understood as upfront medical management is recommended [1]. We report a case of a 17-year-old male with atypical presentation of a large prolactinoma with extensive mass effect, unrecognized visual field deficits, and atypical neuroimaging features mimicking other more common childhood suprasellar tumor types in the absence of symptoms of hyperprolactinemia. 

## Case presentation

**Figure 1:**
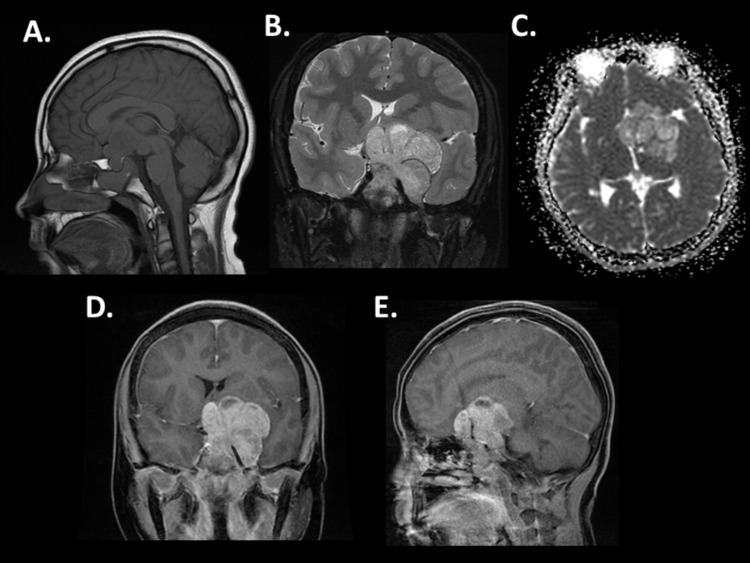
Neuroradiographic features of giant prolactinoma (A) Sagittal non-contrast MRI reveals a large suprasellar mass with obscuration of the optic nerve. T-2 weighted MRI shows extensive lateral invasion (B) and no evidence of reduced diffusivity on apparent diffusion-weighted coefficient sequences (C). Post gadolinium images reveals homogeneous signal with encasement of the cavernous carotid artery (D-E).

**Figure 2:**
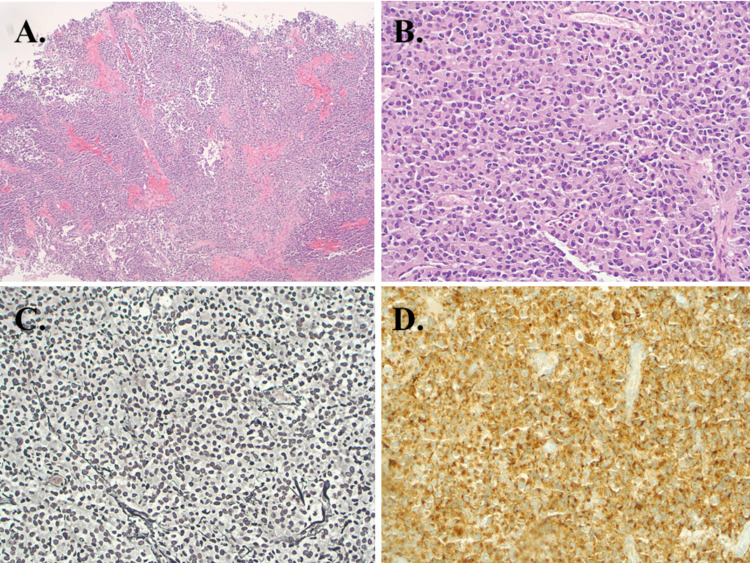
Neuropathologic features of prolactinoma (A) Low power review of tumor demonstrates a solid growth pattern (40X magnification), (B) Tumor cells are relatively uniform with bland appearing nuclei and moderately abundant cytoplasm (200X magnification), (C) Reticulin stain reveals fragmented staining pattern with loss of normal acinar architecture (200x magnification), (D) Tumor cells are diffusely positive for prolactin immunohistochemical stain (20X magnification) consistent with a diagnosis of prolactinoma.

A 17-year-old male presented with six months of headaches, and a few-weeks history of difficulty in seeing the board at school. Neurologic examination at presentation was significant for a right homonymous hemianopsia that was previously unrecognized. MRI revealed a large well-circumscribed multi-lobulated cystic and solid enhancing suprasellar mass with foci of mineralization and no evidence of reduced diffusivity on apparent diffusion coefficient sequences (Figure 1). The mass obscured the optic chiasm and obliterated the left intracranial optic nerve extending into the left Meckel's cave and the left foramen ovale. Radiologic differential diagnosis at the time included craniopharyngioma, and other less likely differential considerations included a low-grade glioma, germinoma, and atypical meningioma. The patient underwent surgical debulking given the extensive mass effect and associated vision loss. Neuropathology demonstrated a relatively monotonous population of cells with mild cytological atypia in focal areas arranged in cords and nests with interspersed capillary blood vessels and focal areas with thin fibrous septa (Figure 2). The tumor cells showed moderate amounts of eosinophilic cytoplasm and round-to-oval shaped nuclei with occasional nucleoli. Reticulin stain revealed fragmented staining pattern with loss of normal acinar architecture. Ki-67 revealed a low proliferative index of 2-3%. Prolactin (PRL) immunohistochemical stain was diffusely positive consistent with a PRL-secreting pituitary adenoma, correlating with the serum findings. Following surgical debulking, the patient had a transient right-sided weakness with resolution of the hemianopsia. As part of the pre-operative endocrinologic workup performed one day prior to surgery and repeated post operatively, a marked elevation of serum PRL (1980ng/ml) that correlated with the histopathologic diagnosis was revealed. The PRL results were not reported until five days after the surgery was completed as rapid testing was not available at our institution. Post operatively, the patient was started on dopamine agonist (DA) cabergoline with a complete response on MRI and normalized PRL levels over many months. He remains five years disease free with a normal neurologic examination.

## Discussion

Prolactinomas are the most common pituitary adenoma presenting most frequently in the age group of 20-50 years with an approximate 10:1 female to male ratio [1]. However, pituitary adenomas are extremely rare in childhood and comprise less than 2% of all intracranial tumors, half of which represent prolactinomas [2]. Although most prolactinomas are spontaneous, considerations should include genetic causes, specifically multiple endocrine neoplasia-I (MEN-I) with a prolactinoma occurrence in 22% of patients and Carney complex patients. Mutations in MEN-1 and Carney complex have been implicated in aryl hydrocarbon receptor-interacting protein familial isolated pituitary adenomas (AIP-FIPA) presenting prolactinoma predominance [1].

Prepubescent clinical presentation of a prolactinoma varies, but typically includes headaches, visual disturbances, and growth failure [2]. Distinctions in presentation include a higher frequency of hypogonadism, pubertal arrest, and galactorrhea in females and macroadenoma in males [3]. The mass effect on the optic chiasm in the patient lends itself to this distinction. The patient presented with symptoms atypical of prolactinoma and neuroimaging features mimicked more common suprasellar tumors, which led to a delay in recognition of the giant prolactinoma, potentially avoiding surgery, prior to surgical resection. 

DA, such as bromocriptine and cabergoline, are the primary treatment for prolactinomas regardless of tumor size, patient age, or gender and are effective in controlling PRL levels shown to be proportional to tumor size and typically result in tumor size reduction that may minimize or even obviate the need for surgery [4]. Surgical intervention as treatment should be considered if there is an acute threat to immediate patient health and is typically followed by DA therapy. Bromocriptine is effective in controlling PRL levels, which are usually proportional to tumor size, and promotes tumor shrinkage. Exceptions are seen in the treatment of macroadenomas with varying results. Although prolactin levels may correlate with tumor size, they do not correlate well with symptoms of hyperprolactinemia until higher PRL levels are reached [5-8].

Cystic macroprolactinomas causing neurological symptoms such as visual field defects have shown to have poor response against DA therapy and are an indication of early surgical intervention [1,4,9]. Lack of evidence for medical therapy as the primary approach for compressive cystic prolactinomas further prompts surgical intervention, followed by DA therapy [5,9]. It is possible that if rapid prolactin testing were available at our institution, upfront medical management may have been initiated prior to debulking.

Bromocriptine is effective in controlling PRL levels, which are usually proportional to tumor size, and promotes tumor shrinkage. Exceptions are seen in the treatment of macroadenomas with varying results. Although PRL levels may correlate with tumor size, they do not correlate well with symptoms of hyperprolactinemia until higher PRL levels are reached [6,10].

Comparison of DA therapy effectiveness pre- and post-surgery is unreliable as primary DA therapy patients present with little to no visual defects that would prompt early intervention [5]. Our case further highlights the importance of visual field examinations as they may often go unrecognized [11].

## Conclusions

Our case highlights successful DA therapy post early interventional surgery for cystic prolactinoma in a patient with progressive visual defect. In most cases of suprasellar tumors, baseline endocrinologic studies are obtained as neuroradiographic features of suprasellar prolactinomas may mimic more common tumor types in this location and symptoms of hyperprolactinemia may be absent despite correlation of PRL levels with prolactinoma size, which may lead to change in management. Furthermore, pre-operative endocrinologic workup may potentially obviate surgical intervention in favor of DA treatments. Macroadenomas with extensive mass effect resulting in neurologic defects are exceptions in which surgical debulking prior to DA treatment may be required for successful pharmacologic therapy.
